# IMPLEMENTATION RESEARCH: INTERGENERATIONAL PROGRAMS ACROSS UNIVERSITY SETTINGS

**DOI:** 10.1093/geroni/igac059.1051

**Published:** 2022-12-20

**Authors:** Jill Juris, Skye Leedahl, Natalie Douglas

## Abstract

Implementing intergenerational programs within university settings has been associated with benefits for all generations involved, which often includes young children, university students, and older adults. However, from conceptualization to pilot testing to evaluation, challenges and opportunities present themselves. This symposium will highlight implementation realities for intergenerational programs within higher education settings. This symposium will specifically address dimensions of: geography (rural versus urban), modality (such as in-person, virtual, or a mix), community/university partnerships, and scholarship for faculty balancing instruction and research demands. Addressing stages of implementation, the papers reflect a continuum from pre-planning to pilot to more advanced stages of implementation. First, Lisa Borrero will highlight challenges and opportunities of conceptualizing fully online intergenerational programming, including the pre-implementation planning, execution, and evaluation stages.The second paper from Ladan Ghazi Saidi will describe pre-implementation tasks completed to establish interest in intergenerational programming in a rural setting, as well as challenges stemming from the pandemic. Third, Jill Juris and colleagues will highlight an online intergenerational technology program offered from a rural Western North Carolina university that began implementation during the pandemic.Fourth, Rachel Scrivano and colleagues will describe a community-based participatory research method that bridged the gap between expectations and reality of implementing a 5-year intergenerational program focused on healthy food access. The fifth paper from Skye Leedahl and colleagues will discuss the implementation experiences of an intergenerational, reverse mentoring, technology program that has evolved for seven years at a Rhode Island public university and utilizes community partners from across the state.

